# Temporal and spatial characterisation of protein liquid-liquid phase separation using NMR spectroscopy

**DOI:** 10.1038/s41467-022-29408-z

**Published:** 2022-04-01

**Authors:** Jack E. Bramham, Alexander P. Golovanov

**Affiliations:** Department of Chemistry, The University of Manchester, Manchester, M13 9PL UK

**Keywords:** Molecular biophysics, Solution-state NMR

## Abstract

Liquid-liquid phase separation (LLPS) of protein solutions is increasingly recognised as an important phenomenon in cell biology and biotechnology. However, opalescence and concentration fluctuations render LLPS difficult to study, particularly when characterising the kinetics of the phase transition and layer separation. Here, we demonstrate the use of a probe molecule trifluoroethanol (TFE) to characterise the kinetics of protein LLPS by NMR spectroscopy. The chemical shift and linewidth of the probe molecule are sensitive to local protein concentration, with this sensitivity resulting in different characteristic signals arising from the dense and lean phases. Monitoring of these probe signals by conventional bulk-detection ^19^F NMR reports on the formation and evolution of both phases throughout the sample, including their concentrations and volumes. Meanwhile, spatially-selective ^19^F NMR, in which spectra are recorded from smaller slices of the sample, was used to track the distribution of the different phases during layer separation. This experimental strategy enables comprehensive characterisation of the process and kinetics of LLPS, and may be useful to study phase separation in protein systems as a function of their environment.

## Abstract (web summary)

Protein liquid-liquid phase separation is an important phenomenon in biology. Here, the authors demonstrate an approach to characterize the evolution of protein phases in both time and space using a fluorinated probe molecule in NMR spectroscopy.

## Introduction

During liquid-liquid phase separation (LLPS), a homogenous mixture of macromolecules separates into two distinct liquid phases, a ‘dense’ condensed phase enriched with a subset of components, and a ‘lean’ phase depleted of these components. This process is increasingly recognised in biology^1,2^, where it is responsible for the formation of membraneless organelles and condensates, including the nucleolus^3^ and stress granules^4^, but also implicated in a range of diseases, including neurodegenerative diseases^5–7^, cataracts^8,9^, and sickle cell anaemia^10^. LLPS is also an important phenomenon in biotechnology, as a purification and processing technique^11,12^, or as an unwanted physical instability in biopharmaceuticals^13^.

LLPS, sometimes referred to as condensation, manifests itself as the appearance of small dense liquid droplets suspended within a lean phase (microscopic LLPS), which often proceeds to the formation of distinct dense and lean layers in the sample once the droplets become large enough and coalesce (macroscopic LLPS). As the dense and lean phases exist in both scenarios, it is convenient to use the term “layer separation” to refer to this final stage of macroscopic LLPS. The kinetics of these processes, and the effect of different conditions or additives on these kinetics, is of particular interest^14,15^. However, these processes are difficult to study by existing techniques. Light scattering due to the presence of liquid droplets, or fluctuations in density or refractive index often results in opalescent or turbid solutions, rendering quantitative optical approaches challenging^16,17^. Fluorescence microscopy using labelled LLPS components or dyes may report on the radius of droplets, but not the concentration of the two phases^15,18^. Additionally, layer separation adds a complicating spatial component, due to non-uniform distribution of the phases throughout the sample^19^. Therefore, the physical and geometrical constraints of biophysical techniques mean each may be limited to studying one aspect of LLPS, and further characterisation techniques are needed to reach a more holistic assessment, particularly regarding the evolution of the concentration and volumes of the two phases.

Nuclear magnetic resonance (NMR) spectroscopy is a powerful biophysical technique, which has recently been applied to characterise protein LLPS. High-resolution multidimensional approaches have been used to probe molecular interactions prior to biological LLPS, and in isolated lean and dense fractions^20–26^. However, the typical properties of phase separating systems render it difficult to use conventional high-resolution protein NMR to study the actual process of phase separation, and particularly that of layer separation. Firstly, these NMR experiments are slow compared to the typical timescales of LLPS. Secondly, layer separation following LLPS leads to significant density and concentration differences across the sample, i.e., between the upper lean and bottom dense fractions, resulting in distorted or broadened NMR signals due to increased magnetic field inhomogeneity^27^. Finally, increased viscosity in the dense phase typically results in poor NMR signal properties due to slow molecular tumbling and fast transverse relaxation^21,28^.

With these considerations in mind, we propose an approach that uses a fluorinated probe molecule, in combination with spatially-selective and conventional bulk-detection ^19^F NMR, to comprehensively study solution behaviour preceding, during and after protein LLPS. The small fluorinated probe transiently interacts with protein molecules, with each evolving phase giving rise to a distinct NMR signal. Each characteristic signal reflects the properties of each individual phase, particularly phase volume and concentration. Additionally, the general signal properties of the probe, such as relaxation and tumbling, are more amenable than those of the larger macromolecule. Therefore, both phases can be monitored and quantified simultaneously by their characteristic signals. Meanwhile, in spatially-selective NMR, application of a selective radiofrequency pulse during a magnetic field gradient allows investigation of a small defined slice of the sample^29^, resulting in localised NMR spectra with reduced field inhomogeneity^30–32^. Spatially-selective NMR therefore enables characterisation of the spatial distribution of the two phases, particularly during layer separation.

In this study, we demonstrate the potential of this experimental approach to characterise both the temporal and spatial aspects of protein LLPS by applying it to the model protein bovine serum albumin (BSA), which undergoes spontaneous LLPS in the presence of yttrium chloride (YCl_3_) at temperatures above a lower critical solution temperature (LCST)^19,33,34^. We show that 10 mM trifluoroethanol (TFE) as a probe molecule is sensitive to the local protein concentration, and can report on the volume and concentration of the different phases emerging or present simultaneously in the sample. The kinetics of LLPS at different temperatures are then examined by bulk-detection NMR, with the emergence of dense and lean phases monitored over time. On a longer timescale, the process of the two phases separating into distinct layers is monitored and characterised by spatially-selective NMR. We show that a fluorinated probe molecule in combination with bulk-detection and spatially-selective ^19^F NMR spectroscopy is an excellent experimental strategy to assess the process of protein LLPS.

## Results

**Fig. 1:**
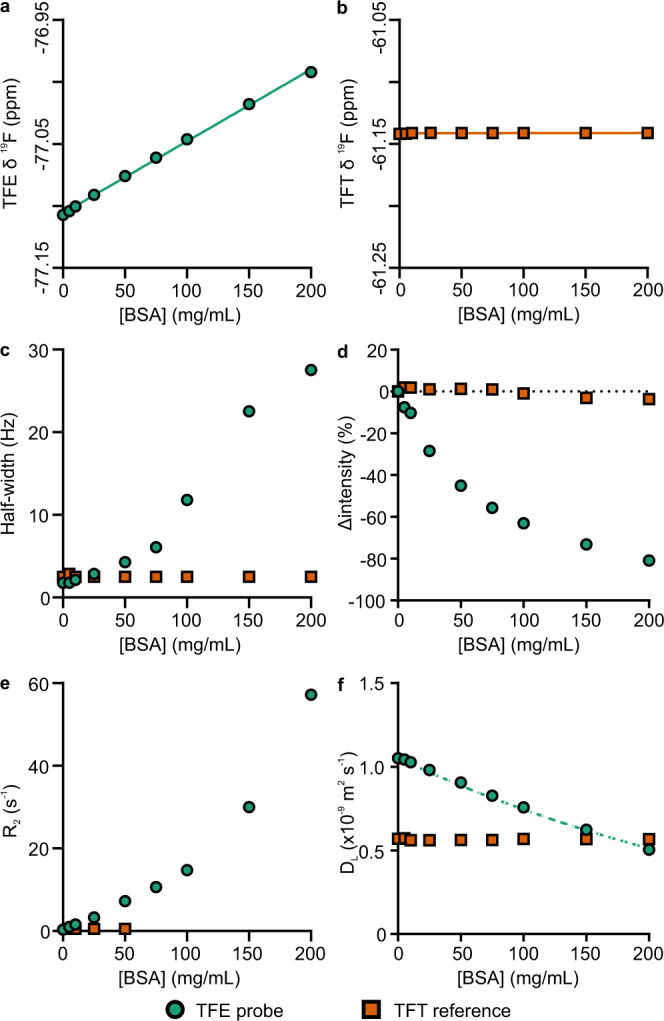
Effect of protein concentration on TFE probe and TFT reference NMR signal parameters. **a** TFE and **b** TFT chemical shift with increasing protein concentration, with linear fits plotted. **c** TFE and TFT linewidth. **d** Change in probe and reference intensities (normalised against intensity in the absence of BSA). Dotted guideline indicates zero level with no change. **e**, **f** Changes in transverse relaxation (*R*_*2*_) and translational diffusion coefficients (*D*_L_), respectively. The dotted guideline in **f** indicates the expected reduction in *D*_L_ as a consequence of increased molecular crowding (Eq. (6)). NMR experiments recorded at 25 °C. For *R*_2_ and *D*_L_ the fitting errors (95% confidence intervals estimated using the Monte Carlo approach) are generally smaller than the symbols used. Source data are provided as a Source Data file.

Here, we investigated the use of a small fluorinated probe molecule, TFE, added to the sample (10 mM final concentration) to study phase transitions and layer separation in a model protein system, BSA, which undergoes LLPS at higher temperatures in the presence of YCl_3_. We found that the TFE ^19^F NMR signal (observed at ∼−77 ppm) is sensitive to protein concentration, as reflected by a number of NMR observable parameters (Fig. 1). Most importantly for our approach, TFE displays changes easily detectable in 1D ^19^F NMR spectra, including linear chemical shift perturbations (δ, Fig. 1a) and increased linewidth (Fig. 1c) with increasing protein concentration. Additionally, TFE δ linearly depends on environmental factors such as temperature and concentration of additives. These changes in ^19^F δ can be used to create a calibration curve under known solution conditions (Eq. (1) and Supplementary Table 1). TFE also displays increased transverse relaxation rate (*R*_2_) and reduced translational diffusion (*D*_L_) with increasing protein concentration (Fig. 1e, f), further suggesting it undergoes transient interactions with protein molecules. The concentration dependence of *D*_L_ closely follows the expected behaviour explained by increased molecular crowding alone (Fig. 1f).

As an additional control, 100 mM trifluorotoluene (TFT) in a coaxial insert in the same sample tube produces a simultaneously observed ^19^F signal (at ∼−61 ppm), while its solvent DMSO-d_6_ provides the deuterium field-frequency lock signal required by the NMR spectrometer, all without any adulteration of the protein sample. This TFT signal is virtually unperturbed by changes in protein concentration (Fig. 1), and its integral and chemical shift can be used as a convenient external reference. Importantly, the lineshape of the TFT ^19^F signal also acts as a sensor for any macroscopic magnetic field inhomogeneity in the outer protein sample, such as that emerging from the process of layer separation. The observable signals from the probe and reference molecules present in the sample therefore report directly on both local protein concentration (TFE) and the macroscopic behaviour of the sample (TFT).

### TFE behaviour in isolated dense and lean fractions

**Fig. 2:**
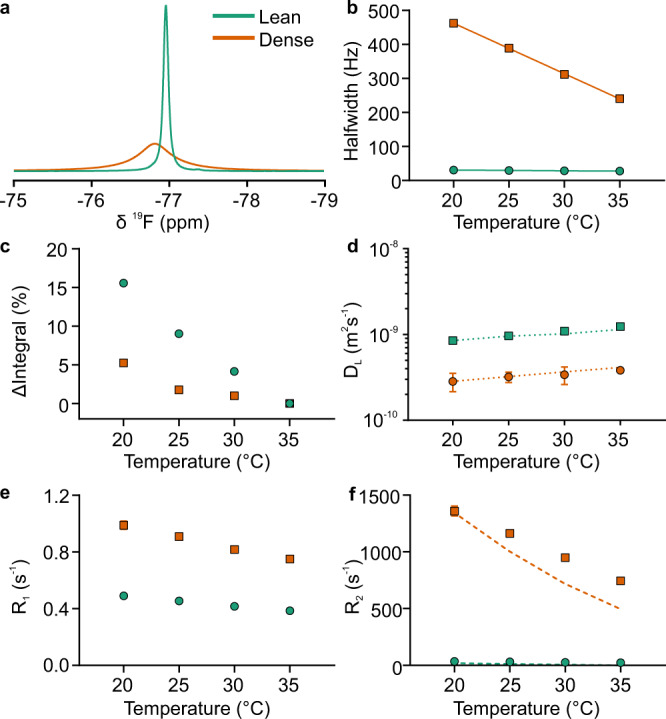
Analysis of the bulk-detected NMR spectra of isolated BSA lean and dense fractions. **a** Superimposed 1D ^19^F NMR spectra of the two fractions acquired at 35 °C. Behaviour of 1D signal line width (**b**) and signal integral normalised against signal at 35 °C (**c**). **d** TFE translational diffusion coefficients with 95% fitting confidence intervals (estimated using the Monte Carlo approach) as measured by DOSY NMR, with dotted lines indicating expected *D*_L_ based on temperature-dependent changes in water viscosity (Eq. (7)). TFE longitudinal (**e**) and transverse (**f**) relaxation rates with 95% fitting confidence intervals as a function of temperature. Dashed guidelines indicate expected behaviour given an assumption that relaxation rate is reduced only due to lower water viscosity and faster molecular tumbling at higher temperature (Eq. (5)). For *R*_1_, *R*_2_ and *D*_L_ the estimated fitting errors are generally smaller than the symbols used. Source data are provided as a Source Data file.

Having established that TFE is sensitive to protein concentration, we next investigated how signal behaviour differs between the dense (360 mg/mL) and lean (80 mg/mL) fractions produced by macroscopic LLPS at 40 °C. LLPS was triggered in protein solutions by temperature incubation, with the resulting dense and lean layers separated, and each of the isolated fractions examined by bulk-detection NMR as a function of temperature (Fig. 2).

Remarkably, despite extremely high concentration and viscosity in the dense fraction, a characteristic signal from the TFE probe is easily detectable in both fractions. These signals were markedly different in the two fractions, with the lean fraction resulting in a narrow intense peak upfield of the broader dense fraction signal (Fig. 2a). The chemical shift difference between the two fractions is consistent with the measured protein concentrations and the calibration relationship established earlier (Fig. 1a). In the dense fraction, TFE exhibited significantly slower diffusion and faster relaxation rates (Fig. 2d–f) than in the lean fraction, likely as a result of increased crowding and TFE transiently interacting with BSA. Together these data in isolated fractions show that TFE is dispersed uniformly throughout the two phases, and not confined to the phase boundaries or edges. Although the temperature dependence of molecular diffusion in both dense and lean fractions can be fully explained by changes in water viscosity (Fig. 2d), R_2_ in the dense fraction decreases with temperature slower than expected from viscosity alone (Fig. 2f), suggesting additional temperature-dependent changes in the chemical exchange regime.

### Tracking fast kinetics of LLPS through bulk-detection NMR

As the lean and dense fractions give rise to distinctive TFE NMR signals, we next explored how NMR can be used to track the simultaneous appearance and development of these phases during the course of LLPS under different conditions. Here, 200 mg/mL BSA with 20 mM YCl_3_ was subjected to temperature jumps from 25 °C to 40, 45 or 50 °C, as a trigger for LLPS. The rate and nature of LLPS were significantly different at increasing temperatures. At 40 and 45 °C, BSA underwent macroscopic LLPS with complete layer separation, while at 50 °C the solution became extremely opalescent, but without subsequent layer separation, suggesting an arrested state (Supplementary Fig. 3).

**Fig. 3:**
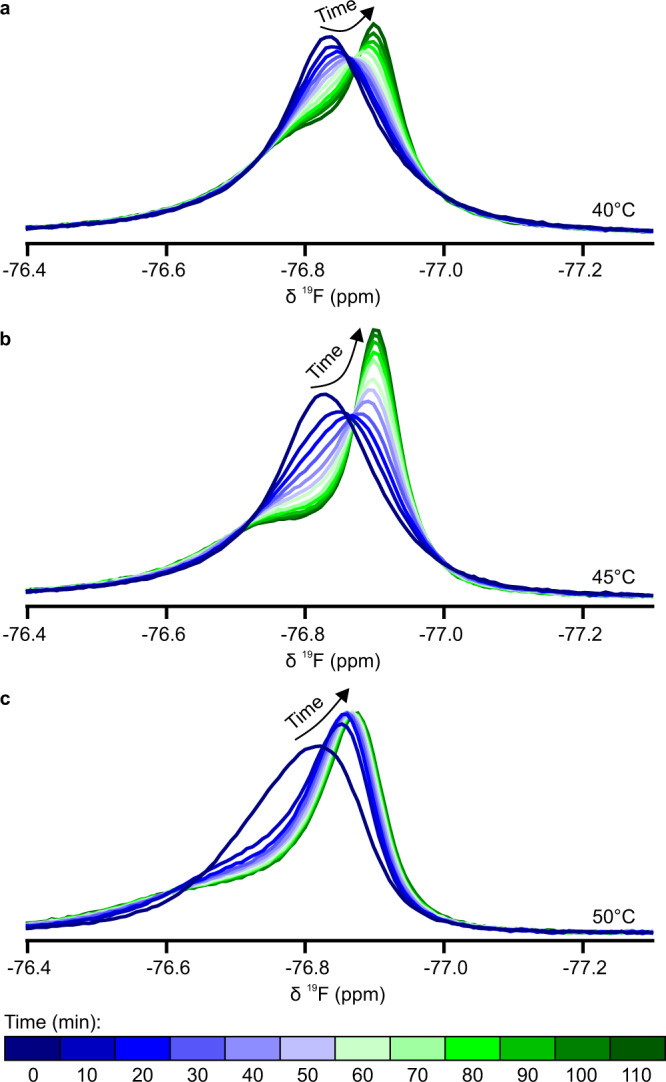
Examples of bulk-detected ^19^F NMR spectra of TFE in BSA undergoing microscopic LLPS at different temperatures following a temperature jump. Spectra at 40 °C (**a**), 45 °C (**b**) and 50 °C (**c**). The representative spectra shown here are sampled at 10 min intervals, from *t* = 0 to 110 min.

For the initial faster kinetics of LLPS preceding layer separation, bulk-detection NMR was used to observe the evolution of the TFE probe signal, and thus the emergence of different phases throughout the sample (Fig. 3). Following the temperature jump trigger, the initial single peak develops into two overlapping species, a narrower upfield peak with a broader downfield shoulder. These species are in good agreement with the TFE signals observed in isolated fractions, with the broad shoulder originating from the dense phase, and the narrow peak originating from the lean phase. Additionally, the chemical shifts, widths and intensities of the species continue to evolve over time, indicating changes in the composition of the two phases during LLPS.

**Fig. 4:**
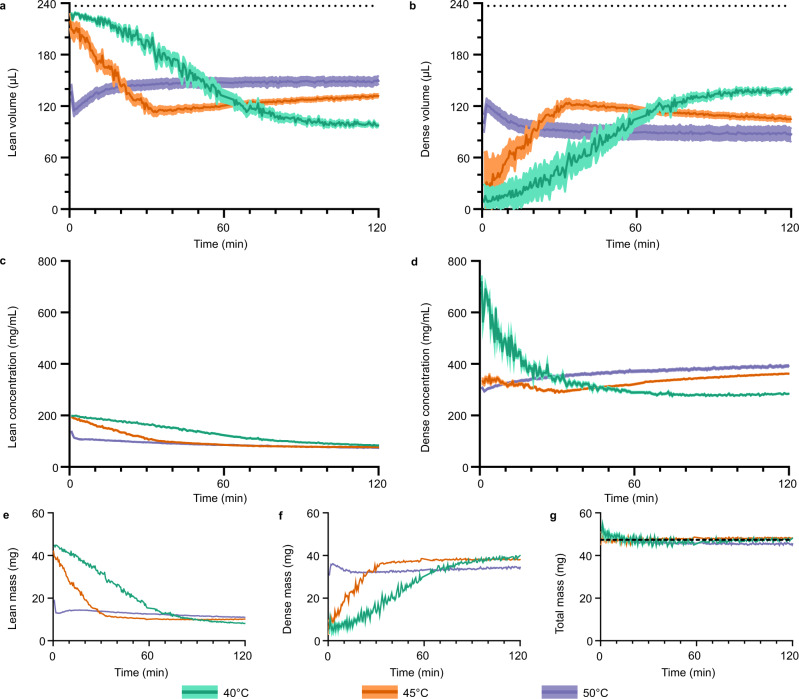
Fast kinetics of BSA LLPS at different temperatures as characterised by bulk-detected NMR. **a**, **b** Apparent volumes of the lean and dense phases, respectively, in the observed sample volume. Volumes were calculated based on deconvoluted integrals of the TFE signals and the known total observed volume (236.8 µL, marked by dotted lines). **c**, **d** Concentrations of the lean and dense phases, respectively. Concentrations were calculated based on deconvoluted peak chemical shifts and protein concentration calibration curve. **e**, **f** Mass of BSA in the lean and dense phases, respectively, present in the observed sample volume. Masses calculated from the apparent volumes and concentrations of each phase. **g** Total mass in the observed volume, based on calculated mass in the lean and dense phases. Dashed line indicates the expected total mass in the observed volume (47.36 mg). For all plots, line plots are the calculated values, while the shaded regions represent parameter error estimates following propagation of 95% confidence intervals in MATLAB fitting. Individual data points represent independent deconvolution results for different time points collected at 0.5 min intervals. Source data are provided as a Source Data file.

To quantify the process of LLPS, TFE probe signals were deconvoluted (Supplementary Figs. 4, 5), revealing two emerging and evolving components, one with lower and another with higher characteristic concentration (Fig. 4). As TFE chemical shift is linearly sensitive to BSA concentration (Fig. 1a), the concentrations of the two evolving species could be determined (Eq. (1), Fig. 4b), while the integral of each signal reports on the apparent volumes of the two species (Eq. (2), Fig. 4a). Together, this determination of the apparent phase volumes and concentrations allows calculation of the observed mass (and hence, population) of protein in each phase (Eq. (3)).

**Fig. 5:**
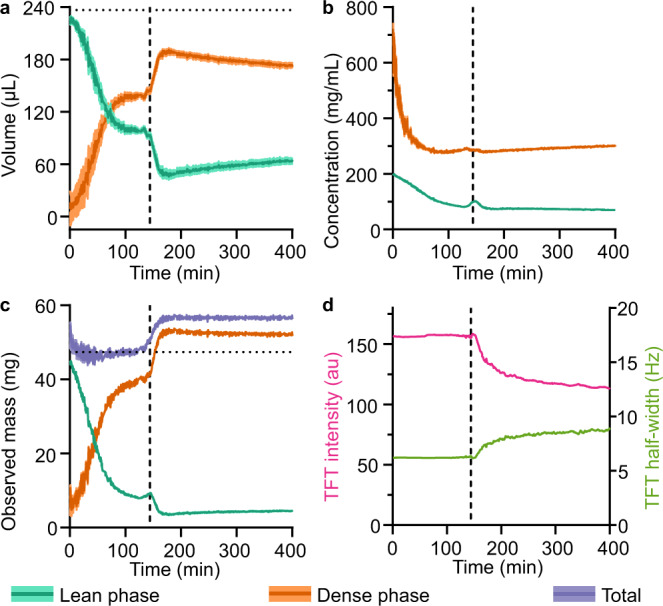
Kinetics of LLPS and layer separation of BSA at 40 °C characterised by bulk-detection NMR. **a** Apparent volume of the lean and dense phases. Dotted horizontal line indicates the observed sample volume (236.8 µL). **b** Concentration of the lean and dense phases. **c** Apparent mass of BSA in the NMR-observed sample volume. Dotted horizontal line indicates the expected total mass in the observed volume in the absence of any layer separation (47.36 mg). **d** TFT reference peak intensity (pink, left axis) and half-width (green, right axis). For all panels, line plots are the calculated values, while the shaded regions represent parameter error estimates following propagation of 95% confidence intervals in MATLAB fitting. Dashed vertical line at 140 min on all plots indicates the onset of layer separation accompanied by decrease in local magnetic field homogeneity. Source data are provided as a Source Data file.

The analysis of data presented in Fig. 4 allows one to measure how the concentration and apparent volume of each phase evolves with time and quantitatively track the process of suspended phases emerging throughout the sample (i.e., microscopic phase separation). Importantly, the experimental results reveal that the kinetics of LLPS in the BSA system at different temperatures goes through the same steps, albeit with substantially different rates. The major similarities include: (i) initial fast drop in concentration (and density) of both emerging lean and dense phases accompanied by rapid increase of the total dense phase volume; (ii) existence of a distinct crossover point where the *minimum* of dense phase concentration and *maximum* volume is reached, followed by (iii) the stage where dense phase compacts, thus increasing its density. Remarkably, the arrested state emerging at 50 °C follows the same trend, with the only difference that dense phase ‘droplets’ are unable to demix and coalesce, and ultimately form a system-spanning network (Supplementary Fig. 3). Additionally, the transition triggered by the lowest temperature, 40 °C, shows evidence of an initial time lag in growth of the dense phase volume, and the crossover time point where the *minimum* dense phase concentration and *maximum* volume is reached is shifted towards the end of the 120 min observation window: such behaviour is more prominently visible for longer observation times, see below and Fig. 5.

Additionally, the concentration of the dense phase after 120 min was also higher at higher temperatures, while the lean phase concentration was very similar at all three temperatures (Fig. 4). Conversely, the volume of the dense phase decreases with temperature (Fig. 4b), such that the final mass of protein in each phase is broadly similar at all three temperatures (Fig. 4e, f). Importantly and reassuringly, at all temperatures, the calculated total protein mass in the observed sample volume remains largely constant and is in good agreement with the expected protein mass (Fig. 4g), which suggests that calibration-based concentration and volume quantification of the emerging phases works well.

### Tracking slower kinetics of layer separation by bulk-detection and spatially-selective NMR

In some systems, LLPS may proceed beyond a suspension of dense droplets and additionally exhibit macroscopic LLPS, with the droplets settling into a lower dense layer with a discrete boundary to an upper lean layer (also see Supplementary Fig. 3)^12,19,21,35^. Therefore, we next extended our experimental approach to study this process of layer separation. Here, the BSA solution previously observed undergoing LLPS at 40 °C was monitored for an extended period, catching both microscopic and macroscopic LLPS (Fig. 5).

After 145 min, the TFE probe exhibited marked changes in behaviour, with a rapid increase in the apparent volume of the dense phase in the observed volume (Fig. 5a) and thus the apparent calculated total mass (Fig. 5c), without significant increase in dense phase concentration (Fig. 5b). These observations arise from layer separation resulting in a significant redistribution of protein across the entire sample volume, with the position of the NMR-observed volume mainly capturing the dense layer towards the bottom of the tube (Supplementary Fig. 3a). Accompanying layer separation, the TFT reference exhibited signs of increased magnetic field inhomogeneity on a macroscopic scale arising from the protein solution, with altered lineshape leading to a reduction in signal intensity and increases in signal width (Fig. 5d). Remarkably, the magnitude of the change in TFT lineshape is very small compared to the broader TFE signal, such that inhomogeneity in this instance does not significantly affect the interpretation of the TFE probe signals.

As layer separation leads to differences in the distribution of the phases across the BSA solution, we next investigated the use of spatially-selective NMR to examine this process in greater detail. Spatially-selective NMR enables signals from a specific horizontal slice of the sample to be collected. Due to non-linearity of the gradient coils required for spatially-selective NMR, the total observable sample length (12 mm/130.1 µL) was less than for bulk-detected NMR (22 mm/238.6 µL) allowing observation of only the central part of the sample (Supplementary Figs. 1, 2).

**Fig. 6:**
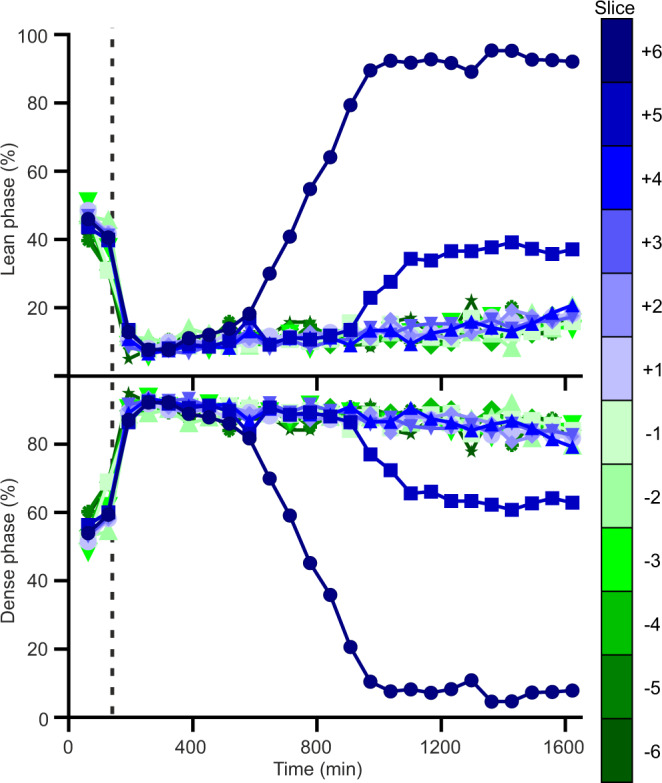
Slow kinetics of BSA LLPS at 40 °C as characterised by spatially-selective NMR. Lean and dense volumes as percentage of total volume in each slice. Dashed vertical lines denote the onset of layer separation at 140 min, as judged by bulk-detection NMR. Source data are provided as a Source Data file.

During microscopic LLPS but before layer separation (Fig. 6, first two time points), each phase is distributed similarly across all slices of the sample (additional data in Supplementary Fig. 6). Additionally, the ratios of the volumes of the two phases detected by spatially-selective NMR are in good agreement with the apparent volumes detected by bulk-detection NMR (Fig. 5). After the onset of layer separation at 140 min, three distinct settling regimes are observed (Fig. 6). Initially, the entire NMR observed volume quickly becomes dominated by the dense phase, which sinks downwards under gravity, with a residual volume of lean phase remaining trapped in this dense layer. At this point, the bulk of lean layer is above the NMR observed volume, while the boundary between the two diffuse layers sinks downwards with time. After 500 min, lean phase population is observed increasing in the top slice (+6) until it is the predominant phase present in this slice after 900 min (Fig. 6), when the boundary is observed moving into the next slice (+5). Analysis of the boundary position reveals that the boundary sinks at a constant rate of 0.154 mm/hr during this regime (Supplementary Fig. 7). Finally, after 1100 min the boundary movement significantly slows, further settling downwards at a rate of 0.004 mm/hr (Supplementary Fig. 7), showing evidence of an even slower process of dense layer evolution, likely linked with gradual release of remnants of lean phase trapped inside the dense layer. Visually, this process corresponds to the reduction of the dense layer opalescence (as initially seen on Supplementary Fig. 3a) with time, typically days, in line with observations with this and other systems undergoing macroscopic LLPS^12,19^. Together these data show that the TFE probe allows one to monitor the entire process of LLPS, from the onset and evolution of the phase transition, through to layer separation and settling, revealing the kinetics and complexities of LLPS.

## Discussion

Phase transitions are seismic events, with the properties of the medium often changing discontinuously and abruptly, and with the kinetics of this process depending on a complex balance of parameters. The nature of protein LLPS makes it a challenging process to study by biophysical techniques, including solution NMR spectroscopy. While conventional high-resolution NMR experiments may be applied under idealised conditions preceding LLPS, or with small amounts of dense droplets suspended in solution, or in isolated fractions^20,25^, applying such techniques to studying the process of LLPS itself has thus far remained extremely challenging. Here, an alternative approach, employing a fluorinated probe molecule with bulk-detection and spatially-selective NMR analysis, is demonstrated as a unique tool to fully characterise the whole process and kinetics of microscopic and macroscopic protein LLPS.

Using a fluorinated molecule which transiently interacts with protein molecules as an NMR probe offers a number of advantages over assessing signals from proteins or other macromolecules themselves^36^. Firstly, fluorine spectra do not suffer from overlapping background signals from biological molecules. Secondly, small molecules have inherently better NMR signal properties than nuclei in proteins, including narrower lineshapes and stronger signal intensities, meaning that their signals are detectable even under challenging conditions, such as in the dense phase, or when recording spectra from a small slice during spatially-selective NMR. Finally, signals from probe molecules may be recorded with a few scans, allowing observations of kinetics at significantly faster timescales. Other small molecules, such as sodium ions in ^23^Na NMR or ammonium ions in ^14^N NMR^37,38^, have also been shown to have potential uses as NMR probes for studying biological condensates or phase separation.

Critically for our approach, the ^19^F chemical shift of TFE exhibits a linear dependency with increasing local protein concentration due to apparent fast exchange between free and BSA-interacting TFE. This regime corresponds to the initial linear part of the receptor-ligand binding isotherm, and is typical for non-specific ligand interactions. As different protein phases emerge during LLPS, the phase boundaries result in slow TFE exchange between phases, giving rise to distinct ^19^F NMR signals whose integrals and chemical shifts can be used to determine the apparent volume and concentration of the two phases. As we measure only the total volume of the phases, the size of individual droplets cannot be directly ascertained, meaning that the final coarsening and coalescence of droplets cannot be monitored as their total volume will be largely static. From a TFE signal viewpoint, there would be no difference in the spectral signature of two smaller separate droplets, or the same droplets merged into one larger droplet.

Another fluorinated molecule (TFT) in a coaxial insert acts as an external reference^39^, and enables observation of magnetic field inhomogeneity emerging as a result of macroscopic layer separation, without the complication of the reference solution itself participating in LLPS. The coaxial insert is also crucial for housing deuterated solvent for field-frequency lock required by the NMR spectrometer, thereby avoiding the addition of ^2^H_2_O directly to the protein solution, which is known to alter the LLPS propensity of proteins^33,40,41^. Despite the presence of two distinct phases emerging during the initial microscopic phase separation, magnetic field homogeneity, as judged by TFT signal lineshape, is initially remarkably unperturbed. After the onset of layer separation, macroscopic field homogeneity degrades, although in this instance, the additional broadening of the TFE signal is very small compared to its linewidth (Fig. 5). Here, spatially-selective NMR is particularly useful, reporting on the distribution of the phases across the sample. Three distinct settling regimes were observed: (i) initial rapid droplet sedimentation, which establishes the diffuse layers; (ii) settling and coalescence of the dense layer; and (iii) a slow process of residual lean phase escape from the dense layer. Spatially selective NMR detection is increasingly recognised as a powerful approach to study a range of complex phenomenon by NMR^31,42,43^, and is possible in all modern NMR spectrometers with Z-gradient coils. It may also be applied to study individual layers in a heterogeneous sample after layer separation, without the need for further sample handling which may affect equilibrium states. Spatially-selective NMR has previously been used to study liquid-liquid interfaces^44,45^ and phase separation in oil mixtures^46^, but to our knowledge has not been applied to protein LLPS.

For our experimental approach to work for other protein systems one needs to establish that the TFE ^19^F chemical shift of the two final fractions is significantly different to allow for spectral deconvolution, meaning that it is best suited to systems where the dense and lean phases have markedly different protein concentrations: this can be established when obtaining calibration curves. For other systems, such as in vitro models of membraneless organelles containing proteins, RNA, and other molecules^20,21^, our experimental approach should still hold, but this would need to be determined experimentally. As NMR imaging experiments may be largely uninformative in organelle systems not exhibiting layer separation, and given that phase separation may inherently occur at much lower concentrations, we anticipate that a much lower probe concentration may be used in such systems, perhaps closer to equimolar, with this only limited by experimental sensitivity in 1D ^19^F NMR. Additionally, other fluorinated small molecules, such as those previously reviewed for other applications^47^, may be superior probes for other systems, selected based on their sensitivity to the concentration gradient. As with the addition of any sample component, care should be taken to ensure that the probe molecule does not alter LLPS behaviour, including transition temperature, conditions needed to trigger the transition, or equilibrium concentration of separated phases. Although TFE is known to stabilise protein alpha helices, the concentration employed here (10 mM or 0.1% v/v) is significantly beneath that shown to perturb BSA conformation^48,49^, and we have not observed any significant effects of TFE on BSA LLPS.

**Fig. 7:**
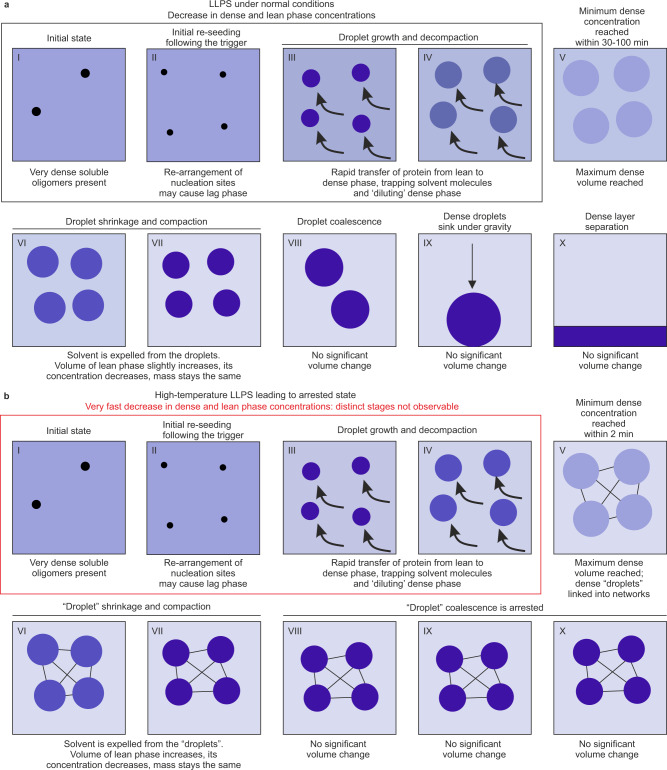
Proposed model for BSA LLPS stages. Progression of LLPS following temperature jumps to 40 °C and 45 °C (**a**), and 50 °C leading to an arrested state (**b**). Intensity of the colour reflects the concentration of the evolving dense and lean species. The shaded areas reflect the total volume of dense and lean phases. The term “droplet” is defined here in a wide sense, as a portion of the sample dense phase characterised by the uniform concentration and density, with the total phase volume known for the whole sample. The size of the individual droplets is not defined here, but for convenience can be assumed to be uniform. For arrested states such “droplets” can be represented as linked networks, preventing their coalescence and settling, whereas for non-arrested states these would correspond to dense phase droplets as observed in solution.

Kinetics of phase separation have previously been widely studied in polymer and colloidal systems^50,51^, and BSA LLPS observed here qualitatively concurs with the classical theories of LLPS derived from these experiments, with distinct nucleation, growth, and droplet settling clearly detected by our parameters. Here, the relatively slow phase separation and noticeable lag period observed at 40 °C suggests a metastable phase transition with dense phase evolution by nucleation and growth^52^. Conversely, the rapid phase separation at 50 °C is consistent with spinodal decomposition. Importantly, here, we directly observe that the concentrations of the lean and dense phase evolve throughout the process, which, to our knowledge, has not been previously described or incorporated in theoretical LLPS models. We propose a mechanistic model for BSA phase separation (Fig. 7), with the dense and lean phases evolving through a set of similar identifiable stages. Initially, highly soluble dense oligomers are present, which likely act as nucleation sites. With time, both dense and lean phase concentrations decrease, while dense phase volume increases at the expense of the lean phase. Material is transferred from the lean phase to the dense phase, resulting in the dilution of the initially extremely highly concentrated dense species. At all temperatures, we observe a distinctive crossover point (Fig. 7a, b, stage V), when the emerging dense phase reaches its minimum concentration but maximum volume. After this point, dense phase concentration starts to increase, while its volume decreases, indicating dense phase shrinkage and compaction. We speculate that shrinkage is caused by the expulsion of the excess solvent trapped during the initial stage of rapid growth of dense phase, with this escaping solvent now essentially diluting the lean phase. Such shrinkage and compaction are expected to be most efficient for smaller droplets having higher surface-to-volume ratio. However, if the competing process of final droplet coarsening and coalescence prevails, then layer separation may occur before shrinkage and compaction is complete (e.g. at 40 °C). Droplet coalescence and coarsening are undetectable by our method as the total volume of dense phase will not be changing, but once droplets become sufficiently large and dense, they will no longer be suspended in solution and instead settle under gravity (Fig. 7a). Conversely, at 50 °C, arrested phase separation occurs (Fig. 7b), with a system-spanning gel-like network formed (Supplementary Fig. 3b). The arrested networked “droplets” are unable to coalesce, preventing layer separation. In another possible scenario not observed here, total dense phase volume may be insufficient for final droplet growth by coalescence, or the dense droplets may not reach sufficiently high density, and no settling and macroscopic layer separation will be observed. This endpoint may be typical for native cellular systems exhibiting LLPS behaviour, such as forming membraneless organelles.

Previous studies have examined the process of BSA LLPS by neutron and X-ray scattering, although at higher YCl_3_ concentrations (~40 mM) leading to faster observed kinetics, with monitoring of the characteristic length parameter showing t^1/3^ dependence consistent with coarsening by diffusion or coalescence mechanisms^19,53^. Although our approach cannot measure droplet size directly, attempts to use total dense mass or volume as proxies for this parameter reveal that the system here exhibits broadly similar apparent kinetics, scaling according to t^1/3^ at lower temperatures, with the exponent decreasing at higher temperatures (see Supplementary Fig. 8 for more details). Furthermore, the final arrested dense phase concentration here appears to follow the dense branch of the binodal^19,54^, rather than intersecting at a lower concentration^53^.

This ability of our NMR approach to detect the apparent volume and protein concentration of each phase, even under challenging experimental conditions, may be combined with other complementary techniques (e.g. fluorescence microscopy, or neutron and X-ray scattering) which examine changes in characteristic lengths or droplet sizes in the evolving system, to provide a holistic understanding of the kinetics and process of protein LLPS. New theoretical models of LLPS would be required to take into consideration the changing concentration and density inside the dense phase as a time dependent process, which may improve our understanding of the early nucleation process as well as droplet coalescence, coarsening and maturation.

This combination of bulk-detection and spatially-selective analysis, with a fluorinated probe molecule, is uniquely suited to studying the dynamic processes of phase and layer separation by NMR spectroscopy. The ability to monitor the emergence and evolution, particularly the concentration and total volume, of both phases simultaneously in situ, with temporal and spatial resolution, makes this approach well-suited to studying the effects of different factors on the composition of the phases and the kinetics of LLPS. Finally, in systems undergoing layer separation, spatially-selective NMR enables tracking of the distribution of the phases and layers throughout the sample. This method of monitoring LLPS kinetics may also be applicable in other model systems, such as intrinsically disordered proteins, where the effect of amino acid substitutions or inclusion of RNA cofactors on LLPS propensity could be investigated^55,56^.

## Methods

### Sample preparation

Lyophilised bovine serum albumin (BSA) powder (essentially fatty acid and globulin free, >99%, Sigma-Aldrich, #A0281) was dissolved in distilled water (Mili-Q) to 10–20 mg/mL, then filtered with 0.1 µm syringe filters (Millex-VV, Merck). BSA solutions were subsequently concentrated by ultrafiltration (Vivaspin 20, 10 kDa MWCO, Sartorius) to the required concentration, with protein concentration measured by absorbance at 280 nm (A_280_) with a NanoDrop (Thermo Scientific) following appropriate dilution. Yttrium chloride (YCl_3_) (99.99%, Sigma-Aldrich) was also dissolved in distilled water, and then filtered with a 0.1 µm syringe filter.

LLPS was studied in protein solutions containing 200 mg/mL BSA with 20 mM YCl_3_ and 10 mM TFE (Sigma-Aldrich). For NMR experiments examining LLPS in situ, 480 µL solution was pipetted into an NMR tube, with the coaxial insert inserted prior to LLPS. For NMR experiments involving bulk assessment of the separate layers, 1 mL protein solution was pipetted into an NMR tube, with LLPS and layer separation triggered in the tube by incubation at 40 °C for 48 h. After centrifugation with a hand crank tube centrifuge, the majority of the lean layer at the top was then transferred to another tube. Finally, the residual lean layer and a small volume of the dense layer were discarded. Coaxial inserts were then inserted into the NMR tubes.

### NMR Spectroscopy

NMR experiments were conducted using a Bruker 500 MHz (470 MHz for ^19^F) Avance III spectrometer with a QCI-F cryoprobe with cooled ^1^H and ^19^F channels, sample temperature control unit, and Z-gradient coils. Solutions containing the TFE probe molecule were placed in standard 5 mm O.D. NMR tubes (Wilmad), with coaxial inserts (50 mm stem length, Wilmad) containing deuterated dimethyl sulfoxide (DMSO-d_6_) (99.8%, Eurisotop) for NMR field-frequency lock and 100 mM α,α,α-trifluorotoluene (TFT) (Sigma-Aldrich) as an external reference and a probe for magnetic field homogeneity (see Supplementary Fig. 1 for schematic). For all experiments, NMR tubes were positioned such that the centre of the sample volume aligned with the centre of the NMR probe coil region. Magnetic field shimming was performed on the initial homogenous sample, with no further shimming between kinetic experiments.

For NMR with bulk detection (i.e., the conventional way of observing signals from the entire sample), 1D ^19^F NMR spectra were recorded with the standard zg Bruker pulse sequence. Kinetic experiments were acquired at 30 s intervals in a pseudo-2D fashion, with the zg2d pulse sequence and processed with a 5 Hz exponential multiplication (EM) window function. All NMR spectra were initially processed in Topspin 4.0.8 (Bruker), with spectra plotted in Prism 8 (GraphPad). Processed spectra were exported in ASCII format, and further analysed, including peak picking, integration, and lineshape analysis, using in-house scripts (MATLAB R2021a). When required, peak deconvolution was also performed on each spectrum independently using in-house MATLAB scripts, with two Lorentzian lineshapes fitted to the experimental data using nonlinear least-squares fitting, with the errors defined as nonlinear data fitting errors (95% confidence intervals).

Phase concentrations were determined based on chemical shift (δ), and the calibration relationship:

1 $$\delta ={\delta }_{0}+{\theta }_{C}{{{{{\rm{C}}}}}}+{\theta }_{T}\Delta {{{{{\rm{T}}}}}}+{\theta }_{Y}{{{{{\rm{Y}}}}}}$$

where ${\delta }_{0}$ is the chemical shift of TFE alone at the reference temperature (25 °C), *C* is protein concentration, Δ*T* is temperature change relative to the reference, *Y* is concentration of an additive (here, YCl_3_), and ${\theta }_{C}$, ${\theta }_{T}$, and ${\theta }_{Y}$ are the measured calibration coefficients for each variable, respectively (see Table S1). Phase volumes (${V}_{P}$) were determined based on the deconvoluted TFE integral in each phase (${I}_{P}$):

2 $${V}_{P}=\frac{{I}_{P}}{{I}_{T}}{{{{{{\rm{V}}}}}}}_{O}$$

where ${I}_{T}$ is the total TFE integral and ${V}_{O}$ is the NMR-observed volume. Apparent total protein mass in each phase (${M}_{P}$) was determined from the NMR-derived phase concentrations (${C}_{P}$) and volumes (${V}_{P}$):

3 $${M}_{P}={C}_{P}{V}_{P}$$

### Spatially selective NMR

Spatially-selective NMR experiments were acquired by selective excitation applied during a magnetic field gradient, with the separate spectra observed for different areas of the same sample along the vertical z-axis. Here, a gradient field strength of 42.4 G/cm was applied concurrently to a G4 cascade selective pulse of bandwidth 16793 Hz, resulting in 1 mm wide excited slices. 12 evenly spaced selective pulse offsets, ranging from −93404 to +93404 Hz, were used, leading to twelve 1 mm slices collected, centred at −5.5 to +5.5 mm from the centre of the gradient coil (Supplementary Fig. 1). This central region exhibited acceptable gradient linearity (as judged by TFT reference signal integrals in spatially-selective spectra), with slice signal intensity corrected during processing to account for any non-linearity (Supplementary Fig. 2). Spatial experiments were acquired in an interleaved manner to enable use of a minimal relaxation delay (D1) of 0.1 s. Spatially-selective NMR series were initially processed in Topspin 4.0.8 with a 20 Hz EM window function, with auto-phase (apk0) and auto-baseline correction (absn), followed by spectral deconvolution using MATLAB.

### Relaxation

^19^F longitudinal relaxation times (*T*_1_) were measured using the standard inversion recovery sequence (t1ir) with 8 time points, while transverse relaxation times (*T*_2_) were measured with a version of the Bruker Carr-Purcell-Meiboom-Gill (CPMG) sequence with perfect echo using 16 time points. CPMG echo time and repeats were altered to suit individual experimental conditions. *T*_1_ and *T*_2_ were calculated in Dynamics Center 2.5 (Bruker), with the errors defined as nonlinear data fitting errors (95% confidence intervals). Assuming that the transverse relaxation rate (*R*_2_) is reduced only due lower viscosity and faster molecular tumbling at higher temperatures, the expected behaviour of *R*_2_ at higher temperatures was calculated based on the proportionality of *R*_2_ to rotational correlation time (${\tau }_{{{{{{\rm{C}}}}}}}$), which can be derived from the Stokes–Einstein–Debye equation:

4 $${R}_{2}\propto {\tau }_{{{{{{\rm{C}}}}}}}=\frac{4\pi \eta {({r}_{{{{{{{\rm{eff}}}}}}}})}^{3}}{3{kT}}$$

where *η* is dynamic viscosity, *r*_eff_ is the effective radius of the species, *k* is the Boltzmann constant, and *T* is temperature. Rearranging Eq. (4) gives the relaxation rate at temperature *T*:

5 $${R}_{2}^{T}={R}_{2}^{{{{{{{\rm{ref}}}}}}}}\frac{{\eta }_{T}{T}_{{{{{{{\rm{ref}}}}}}}}}{{\eta }_{{{{{{{\rm{ref}}}}}}}}T}$$

where ${\eta }_{T}$ and ${\eta }_{{{{{{{\rm{ref}}}}}}}}$ are the dynamic viscosities of water at temperature *T* and the reference temperature ${T}_{{\mathrm ref}}$, respectively.

### Translational diffusion

Translational diffusion coefficients (*D*_L_) were determined by diffusion ordered spectroscopy (DOSY) using the simulated echo pulsed-field gradient (PFG) pulse program stebpgp1s, using 16 gradient increments. DOSY delays were optimised for each experimental condition, with diffusion times of 150 and 1500 ms, and gradient lengths of 1.5 and 0.3 ms for the lean and dense fractions, respectively. *D*_L_ were calculated in Dynamics Center 2.5, with the errors defined as nonlinear data fitting errors (95% confidence intervals). The expected values of the diffusion coefficient (${D}_{L}^{e}$) at higher protein concentrations were calculated using the value of the diffusion coefficient measured in diluted conditions (${D}_{{{{{{\rm{L}}}}}}}^{{{{{{\rm{e}}}}}}}$) and the model for molecular crowding^57,58^:

6 $${D}_{{{{{{\rm{L}}}}}}}^{{{{{{\rm{e}}}}}}}(\varphi )={D}_{{{{{{\rm{L}}}}}}}^{{{{{{{\rm{diluted}}}}}}}}\times \frac{({1-\varphi })^{3}}{(1+\frac{3}{2}\varphi +{2\varphi }^{2}+{3\varphi }^{3})}$$

where *φ* is the BSA volume fraction in solution, calculated using protein concentrations and a specific volume of 0.735 mL/g for BSA^59^. The expected values of the diffusion coefficient at higher temperature *T* (${D}_{{{{{{\rm{L}}}}}}}^{T}$) were calculated using the rearranged Stokes–Einstein equation:

7 $${D}_{{{{{{\rm{L}}}}}}}^{T}={D}_{{{{{{\rm{L}}}}}}}^{{{{{{{\rm{ref}}}}}}}}\frac{{\eta }_{{{{{{{\rm{ref}}}}}}}}T}{{\eta }_{T}{T}_{{{{{{{\rm{ref}}}}}}}}}$$

where ${D}_{{{{{{\rm{L}}}}}}}^{{{{{{{\rm{ref}}}}}}}}$ is the diffusion coefficient at the reference temperature ${T}_{{{{{{{\rm{ref}}}}}}}}$, and ${\eta }_{T}$ and ${\eta }_{{{{{{{\rm{ref}}}}}}}}$ are the dynamic viscosities of water at temperature $T$ and ${T}_{{{{{{{\rm{ref}}}}}}}}$, respectively.

### Reporting summary

Further information on research design is available in the Nature Research Reporting Summary linked to this article.

## Supplementary information

Supplementary Information

Reporting Summary

## Data Availability

Source data are provided with this paper for Figs. 1, 2, 4, 5 and 6, and Supplementary Figs. 2, 5, 6, 7 and 8. Source data are provided with this paper.

## Source data

Source Data

## Code availability

Code used in the preparation of this study is available from the corresponding author upon reasonable request.
