# Supplementary material for: Temporal and spatial characterisation of protein liquid-liquid phase separation using NMR spectroscopy
Source: Nat Commun. 2022 Apr 1;13:1767. doi: 10.1038/s41467-022-29408-z (PMC8976059; doi:10.1038/s41467-022-29408-z)
Supplement: Supplementary file 1 — Supplementary Information [file 41467_2022_29408_MOESM1_ESM.pdf]

Supplementary Information

Temporal and spatial characterisation of protein liquid-liquid phase separation using NMR spectroscopy

Jack E. Bramham[1] and Alexander P. Golovanov[1*]

[1] Department of Chemistry, School of Natural Sciences, Faculty of Science and Engineering, The University of Manchester, Manchester, UK

*E-mail: a.golovanov@manchester.ac.uk

Table of Contents:

Supplementary Figure 1: Schematic of NMR tube and NMR detection volumes.

Supplementary Figure 2: Effect of slice centre position on spatially-selective NMR signal integrals.

Supplementary Figure 3: Appearance of phase-separated BSA solutions after incubation at 40°C and 50°C.

Supplementary Figure 4: Examples of spectral deconvolution of the TFE ^{19}F NMR signal following LLPS at 45°C.

Supplementary Figure 5: Time dependence of deconvoluted TFE signal parameters from BSA LLPS at different temperatures.

Supplementary Figure 6: Additional deconvoluted parameters from spatially-selective assessment of BSA LLPS.

Supplementary Figure 7: Determination of boundary position and sedimentation rate by spatially-selective NMR.

Supplementary Figure 8. Additional kinetic analysis of BSA microscopic LLPS using dynamic scaling model.

Supplementary Table 1: TFE chemical shift calibration factors

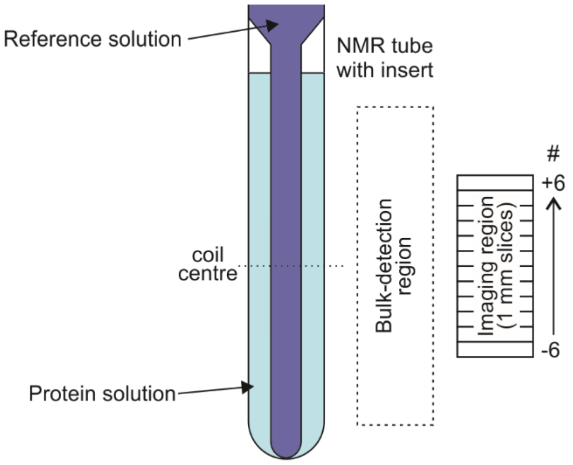

Supplementary Figure 1. Schematic of NMR tube and NMR detection volumes. Protein solution (blue) with TFE probe molecule in standard 5 mm NMR tube, with reference solution (purple) with TFT in DMSO-d6 in coaxial insert inserted into the tube. Selected region for imaging (12 mm length) for spatially-selective NMR in the centre of the bulk-detection region (22 mm). Not to scale.

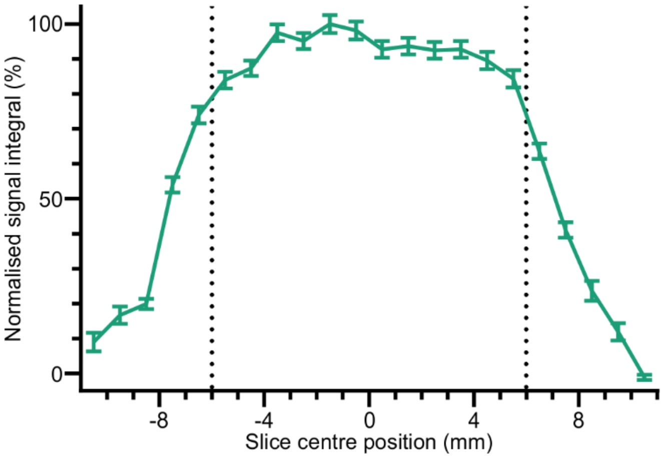

Supplementary Figure 2. Effect of slice centre position on spatially-selective NMR signal integrals. Slice centre position relative to the centre of the gradient coils (e.g. 0 Hz offset). 1 mm slices, centred at 0.5 mm intervals, with positive values above the coil centre, and negative values beneath the coil centre. Error estimates based on spectra signal/noise ratios. Area between the dotted lines represents the region used here in spatially-selective experiments. Outside of this central region, signal integrals falls rapidly (due to non-linearity of the gradient coils) to beneath acceptable levels, particularly when combined with the significantly reduced signal from the small slices. Source data are provided as a Source Data file.

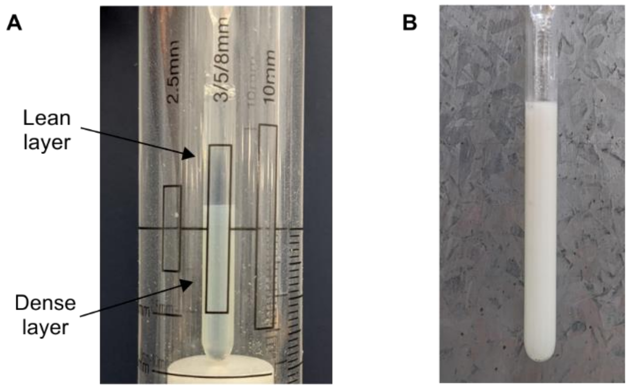

Supplementary Figure 3. Appearance of phase-separated BSA solutions after incubation at 40°C and 50°C. (**A**) Layer separation after incubation at 40°C. Scale from NMR tube depth gauge (1 mm intervals visible on the right-hand side). The dense layer appears opalescent due to incomplete floatation of the lean phase droplets trapped within the dense layer. This opalescence reduces with time as layers settle further, or can be speed up by centrifugation. (**B**) Arrested state after incubation at 50°C. Solutions were incubated for >18 hours, with image taken at room temperature immediately after removal from NMR spectrometer. Appearance of this arrested state was unchanged by cooling, or prolonged refrigerated storage. Remarkably, despite the appearance of this sample, TFE ^{19}F NMR signals are still readily detectable and informative (Figure 4).

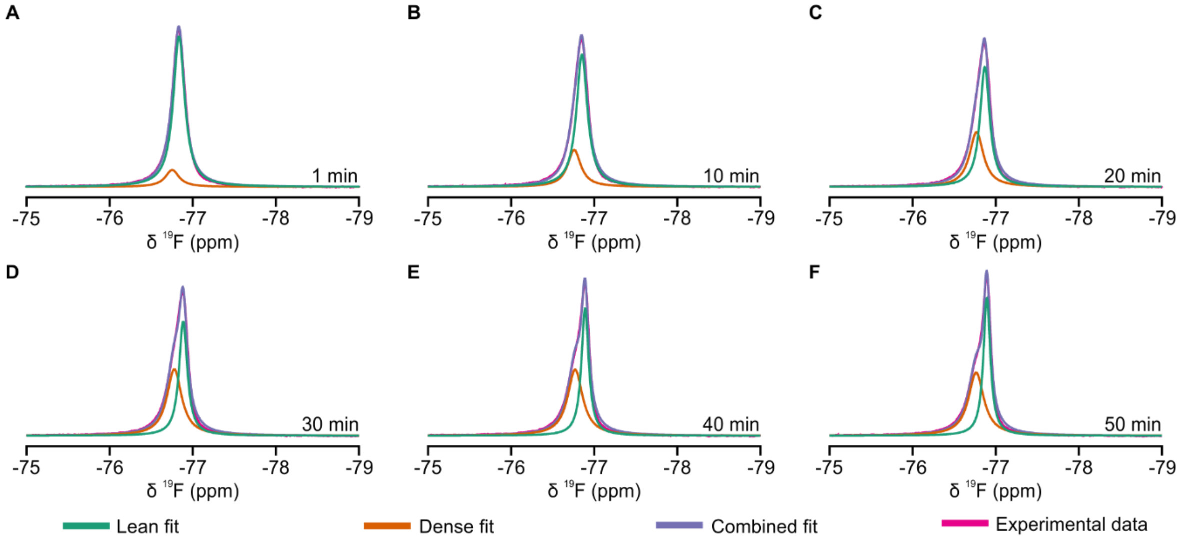

Supplementary Figure 4. Examples of spectral deconvolution of the TFE ^{19}F NMR signal following LLPS at 45°C. Deconvoluted bulk-detected ^{19}F NMR spectra of TFE in BSA at (**A**) 1 min, (**B**), 10 min, (**C**) 20 min, (**D**) 30 min, (**E**) 40 min, and (**F**) 50 min after the temperature jump triggering LLPS. Experimental data (pink) fitted to two Lorentzian lineshapes representing the lean (teal) and dense (orange) phases, with the sum of these two lineshapes as the total combined fit (purple).

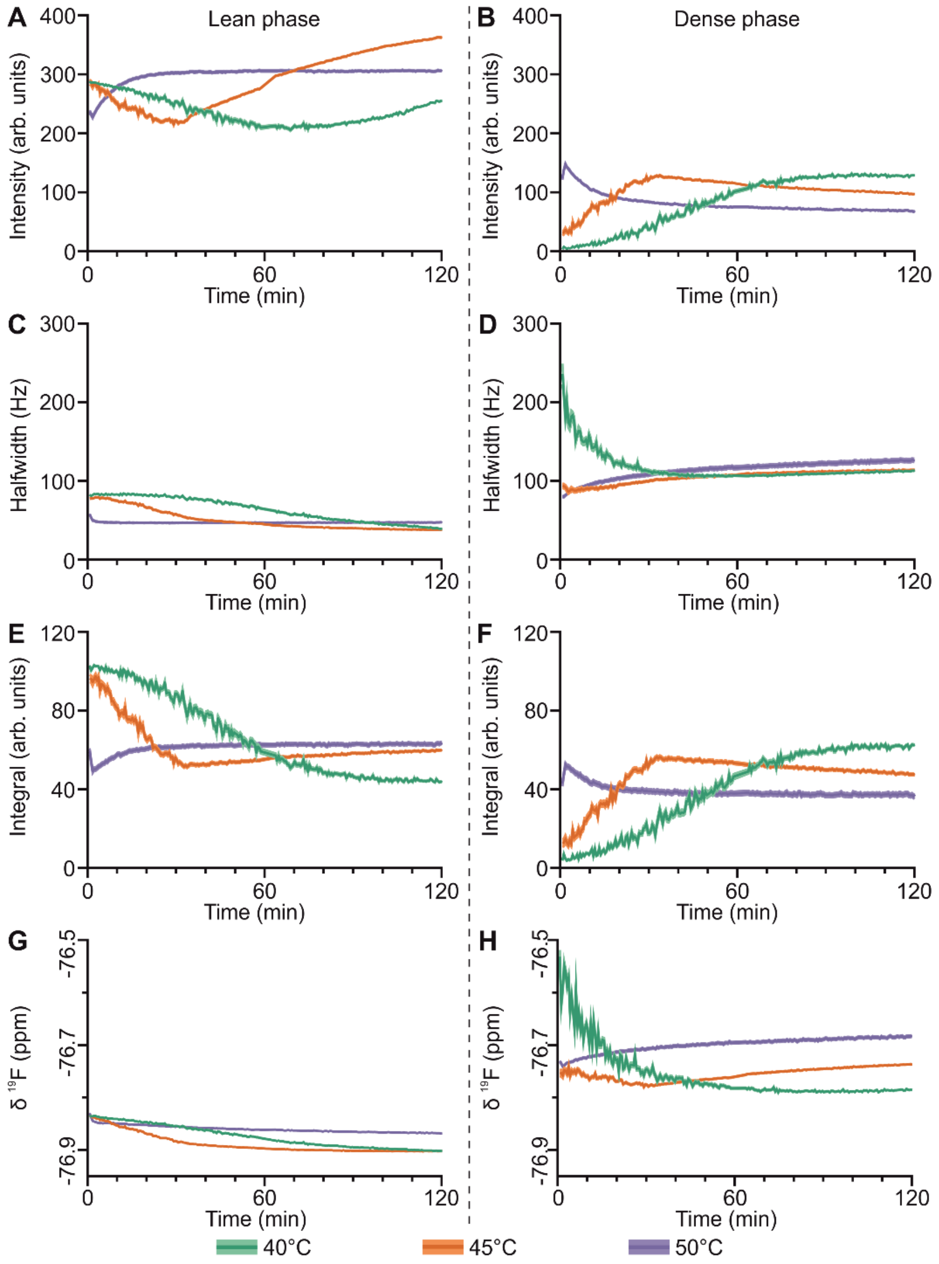

Supplementary Figure 5. Time dependence of deconvoluted TFE signal parameters from BSA LLPS at different temperatures. Left and right columns correspond to lean and dense phases, respectively. (**A-B**) TFE signal intensity. (**C-D**) Signal half-width. (**E-F**) Signal integral. (**G-H**) Signal chemical shift. For all plots, line plots represent the calculated values derived from the deconvolution procedure, while the shaded regions represent 95% confidence intervals reported by the same procedure. Source data are provided as a Source Data file.

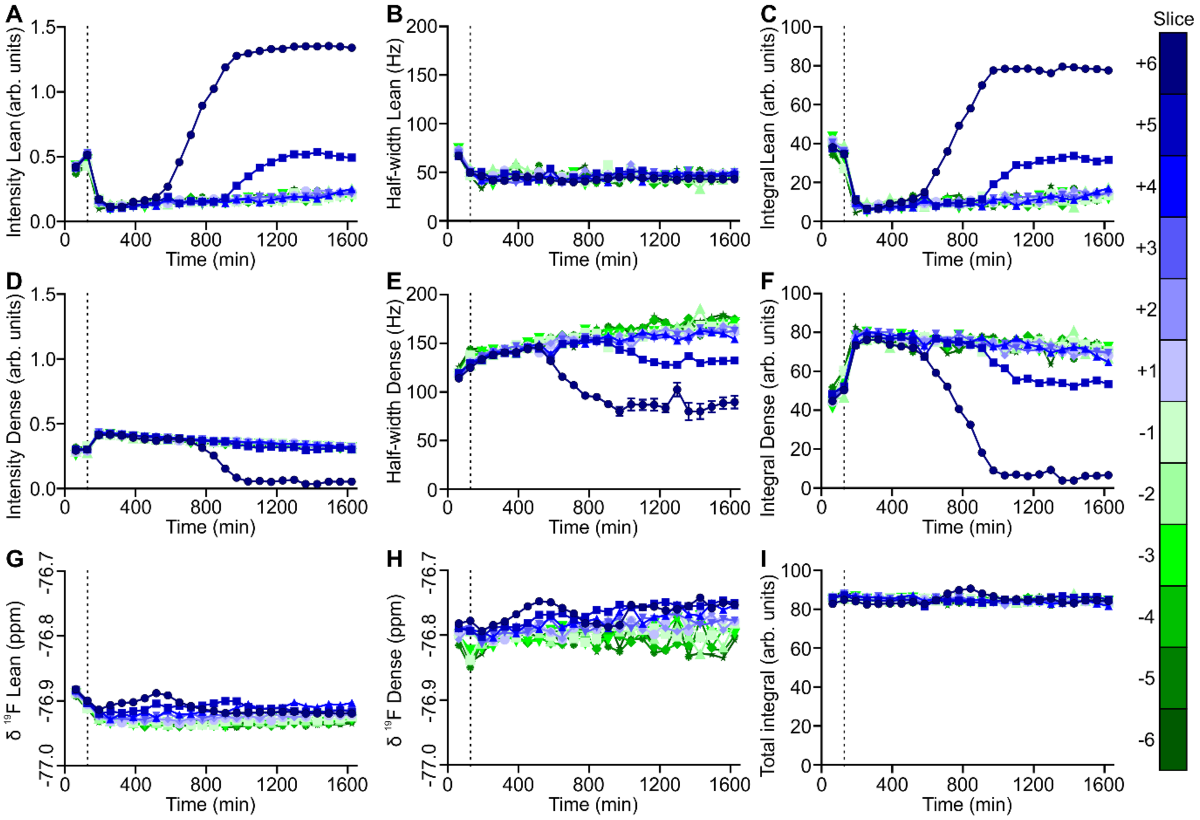

Supplementary Figure 6. Additional deconvoluted parameters from spatially-selective assessment of BSA LLPS. Deconvoluted parameters for the TFE lean phase signal intensity (**A**), half-width (**B**), integral (**C**), and chemical shift (**G**). Deconvoluted parameters for the TFE dense phase signal intensity (**D**), half-width (**E**), integral (**F**), and chemical shift (**H**). Error bars derived from deconvolution procedure and represent 95% confidence intervals. (**I**) Total TFE signal integral. Dashed vertical lines denote the onset of layer separation at 140 minutes, with fast initial kinetics preceding this time, and slower kinetics after this time. Source data are provided as a Source Data file.

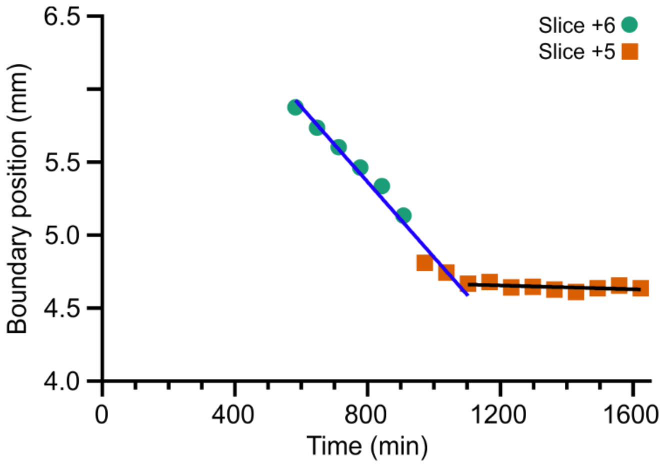

Supplementary Figure 7. Determination of boundary position and sedimentation rate by spatially-selective NMR. Following entry of the lean layer into the spatially-selective detection volume after 500 min, the vertical position of the boundary between the two layers ($Z_{boundary}$) can be determined based on the fraction of dense phase ($Fraction_{Dense}$) in each slice: $Z_{boundary} = Z_{slice\ bottom} + (Fraction_{Dense}\ x\ 1)$, where $Z_{slice\ bottom}$ is the position of the bottom of each slice, and 1 is the height of each slice (in mm). Linear fit to time dependence of the boundary position within slices #+6 and #+5 (blue line) gives a sedimentation rate of 0.154 mm/hr, with second linear fit (black line) representing the final settling of the dense layer, occurring at a rate of 0.004 mm/hr. Source data are provided as a Source Data file.

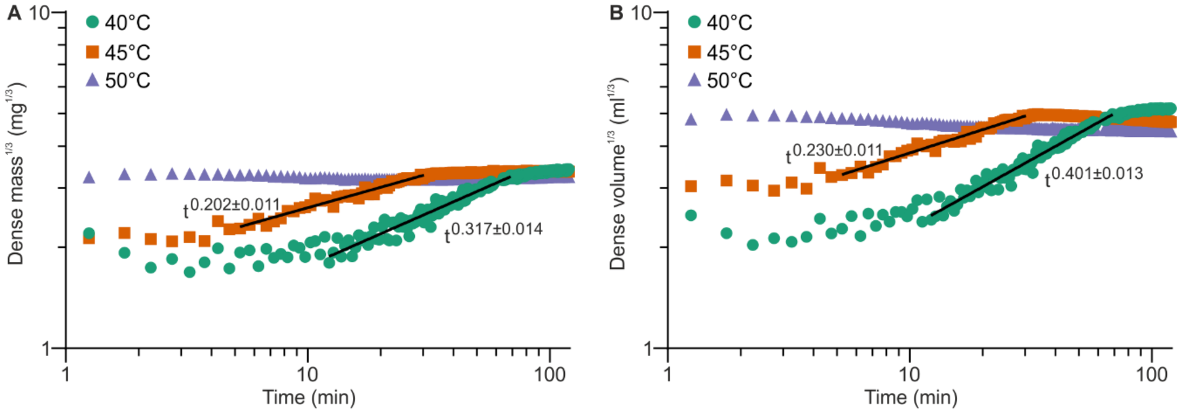

Supplementary Figure 8. Additional kinetic analysis of BSA microscopic LLPS using the dynamic scaling model. Cube root of the mass (A) and volume (B) of the dense phase used as proxies for the characteristic length i.e. droplet radius. Linear fits to experimental data (between 12 and 70 minutes for 40°C, and 5 and 30 minutes for 45°C), with gradients and 95% confidence intervals reported. Source data are provided as a Source Data file. The kinetics of phase separation is often interpreted using the concept of dynamic scaling, formulated on the premise that domain kinetics are controlled by a single characteristic length scale within a given dynamic scaling regime[1,2]. A number of power law functions, and characteristic parameter values, have been suggested to identify the specific growth or coarsening mechanisms at work by fitting the model to the experimental data.[2] Such an analysis of characteristic length scale ξ has been performed for the BSA system based on ultra-small angle X-ray scattering (USAXS) data which revealed that the characteristic length increases with time with a power of 1/3, consistent with growth driven by diffusion or coalescence mechanisms.[3] These simple dynamic scaling models however assume constant concentration and density of the emerging dense droplets, which, as our current study shows, is not always the case. Nevertheless, we plotted the cubic route of dense mass and cubic root of dense volume, as a proxies for characteristic length in our system, as a function of time. Here, these parameters can capture only the initial droplet growth, when the material is transferred from the lean phase into dense, as the later stage of droplet coalescence and coarsening is not accompanied by significant dense mass or volume change. The dense mass parameter exhibits similar behaviour to Ref[3], with the scaling exponent also observed to decrease here with increasing temperature. However, the parameter derived from dense volume did not exhibit this typical behaviour, showing that time-dependent changes in the phase density are important for model interpretation. Therefore new theory developments would be needed to take into account time-dependent density changes and to fit the experimental data obtained here.

Supplementary Table 1. TFE chemical shift calibration coefficients for protein concentration, temperature and YCl_3 concentration terms, respectively, of Equation (1).

	θ_C (ppm·ml/mg)	θ_T (ppm/°C)	θ_Y (ppm/mM)
Calibration factor	0.000580	0.008047	0.000420

Supplementary References

1 Furukawa, H. A dynamic scaling assumption for phase separation. *Advances in Physics* **34**, 703-750, doi:10.1080/00018738500101841 (1985).

2 Berry, J., Brangwynne, C. P. & Haataja, M. Physical principles of intracellular organization via active and passive phase transitions. *Reports on progress in physics. Physical Society (Great Britain)* **81**, 046601, doi:10.1088/1361-6633/aaa61e (2018).

3 Da Vela, S. *et al.* Kinetics of liquid-liquid phase separation in protein solutions exhibiting LCST phase behavior studied by time-resolved USAXS and VSANS. *Soft Matter* **12**, 9334-9341, doi:10.1039/c6sm01837h (2016).
