# Supplementary material for: Temporal and spatial characterisation of protein liquid-liquid phase separation using NMR spectroscopy
Source: Nat Commun. 2022 Apr 1;13:1767. doi: 10.1038/s41467-022-29408-z (PMC8976059; doi:10.1038/s41467-022-29408-z)
Supplement: Supplementary file 2 — Reporting Summary [file 41467_2022_29408_MOESM2_ESM.pdf]

nature portfolio

Corresponding author(s): Alexander Golovanov
Last updated by author(s): Feb 3, 2022

Reporting Summary

Nature Portfolio wishes to improve the reproducibility of the work that we publish. This form provides structure for consistency and transparency in reporting. For further information on Nature Portfolio policies, see our Editorial Policies and the Editorial Policy Checklist.

Statistics

For all statistical analyses, confirm that the following items are present in the figure legend, table legend, main text, or Methods section.

n/a	Confirmed	
☐	☒	The exact sample size (n) for each experimental group/condition, given as a discrete number and unit of measurement
☒	☐	A statement on whether measurements were taken from distinct samples or whether the same sample was measured repeatedly
☒	☐	The statistical test(s) used AND whether they are one- or two-sided<br>*Only common tests should be described solely by name; describe more complex techniques in the Methods section.*
☒	☐	A description of all covariates tested
☒	☐	A description of any assumptions or corrections, such as tests of normality and adjustment for multiple comparisons
☐	☒	A full description of the statistical parameters including central tendency (e.g. means) or other basic estimates (e.g. regression coefficient) AND variation (e.g. standard deviation) or associated estimates of uncertainty (e.g. confidence intervals)
☒	☐	For null hypothesis testing, the test statistic (e.g. F, t, r) with confidence intervals, effect sizes, degrees of freedom and P value noted<br>*Give P values as exact values whenever suitable.*
☒	☐	For Bayesian analysis, information on the choice of priors and Markov chain Monte Carlo settings
☒	☐	For hierarchical and complex designs, identification of the appropriate level for tests and full reporting of outcomes
☒	☐	Estimates of effect sizes (e.g. Cohen's d, Pearson's r), indicating how they were calculated

Our web collection on statistics for biologists contains articles on many of the points above.

Software and code

Policy information about availability of computer code

Data collection	Topspin 4.0.8
Data analysis	MATLAB R2021a; Prism 8 (GraphPad); Dynamics Center 2.5 (Bruker); Excel 2016.

For manuscripts utilizing custom algorithms or software that are central to the research but not yet described in published literature, software must be made available to editors and reviewers. We strongly encourage code deposition in a community repository (e.g. GitHub). See the Nature Portfolio guidelines for submitting code & software for further information.

Data

Policy information about availability of data

All manuscripts must include a data availability statement. This statement should provide the following information, where applicable:

- Accession codes, unique identifiers, or web links for publicly available datasets
- A description of any restrictions on data availability
- For clinical datasets or third party data, please ensure that the statement adheres to our policy

Source data are provided with this paper for Figures 1, 2, 4, 5 and 6, and Supplementary Figures 2, 5, 6, 7 and 8.

Field-specific reporting

Please select the one below that is the best fit for your research. If you are not sure, read the appropriate sections before making your selection.

☒ Life sciences ☐ Behavioural & social sciences ☐ Ecological, evolutionary & environmental sciences

For a reference copy of the document with all sections, see nature.com/documents/nr-reporting-summary-flat.pdf

Life sciences study design

All studies must disclose on these points even when the disclosure is negative.

Sample size

The number of data points was different in different parts of the study. For concentration dependencies eight independently-prepared samples were used, spanning the whole range of protein concentrations , from 5 mg/ml to 200 mg/ml. This sample size was limited by the available experimental time. The experiments presented on Figure 2 were done independently at four different temperatures, using two samples (lean and dense) per temperature. Time series for monitoring kinetics (figure 4) were acquired for 120 with 30 seconds intervals, with two spatially-selective experiments interleaved at the end of each hour creating a time gap in the series, yielding typically 230 independently measured points, at three different temperatures, using three independent samples. For Figure 5 there was 747 independently measured time points. All these spectra were independently deconvoluted, yielding data plotted on Figures 5 and 6. Spatially-selective data was measured independently in 12 slices, as 24 time points.

Data exclusions

No raw experimental data was excluded. On few occasions, for few time points the deconvolution did not converge for unknown reason, in which cases the deconvoluted data were not available. Such cases are labelled explicitly in the Source Data file.

Replication

A number of independently-prepares samples was used - see section "Sample Size". NMR spectra acquired inherently imply repeated measurements via multiple scans and subsequent averaging.

Randomization

Concentration dependencies or Time series were typically observed, and were not subject to randomisation.

Blinding

Blinding was not relevant for the type of direct measurements conducted. The deconvolution of individual spectra was performed by an automated script.

Reporting for specific materials, systems and methods

We require information from authors about some types of materials, experimental systems and methods used in many studies. Here, indicate whether each material, system or method listed is relevant to your study. If you are not sure if a list item applies to your research, read the appropriate section before selecting a response.

Materials & experimental systems

n/a	Involved in the study	
☒	☐	Antibodies
☒	☐	Eukaryotic cell lines
☒	☐	Palaeontology and archaeology
☒	☐	Animals and other organisms
☒	☐	Human research participants
☒	☐	Clinical data
☒	☐	Dual use research of concern

Methods

n/a	Involved in the study	
☒	☐	ChIP-seq
☒	☐	Flow cytometry
☒	☐	MRI-based neuroimaging
